# Finite Element Analysis of Meniscal Anatomical 3D Scaffolds: Implications for Tissue Engineering

**DOI:** 10.2174/1874120700701010023

**Published:** 2007-08-07

**Authors:** L Moroni, F.M Lambers, W Wilson, C.C van Donkelaar, JR de Wijn, R Huiskesb, C.A van Blitterswijk

**Affiliations:** a Institute for BioMedical Technology (BMTI), University of Twente, P.O. Box 217, 7500 AE Enschede, The Netherlands; b Department of Biomedical Engineering, Eindhoven University of Technology, Eindhoven, P.O. Box 513, 5600 MB, The Netherlands

**Keywords:** Rapid prototyping, Mechanical analysis, Finite element analysis, Scaffolds, Meniscus, Tissue engineering.

## Abstract

Solid Free-Form Fabrication (SFF) technologies allow the fabrication of anatomical 3D scaffolds from computer tomography (CT) or magnetic resonance imaging (MRI) patients’ dataset. These structures can be designed and fabricated with a variable, interconnected and accessible porous network, resulting in modulable mechanical properties, permeability, and architecture that can be tailored to mimic a specific tissue to replace or regenerate. In this study, we evaluated whether anatomical meniscal 3D scaffolds with matching mechanical properties and architecture are beneficial for meniscus replacement as compared to meniscectomy. After acquiring CT and MRI of porcine menisci, 3D fiber-deposited (3DF) scaffolds were fabricated with different architectures by varying the deposition pattern of the fibers comprising the final structure. The mechanical behaviour of 3DF scaffolds with different architectures and of porcine menisci was measured by static and dynamic mechanical analysis and the effect of these tissue engineering templates on articular cartilage was assessed by finite element analysis (FEA) and compared to healthy conditions or to meniscectomy. Results show that 3DF anatomical menisci scaffolds can be fabricated with pore different architectures and with mechanical properties matching those of natural menisci. FEA predicted a beneficial effect of meniscus replacement with 3D scaffolds in different mechanical loading conditions as compared to meniscectomy. No influence of the internal scaffold architecture was found on articular cartilage damage. Although FEA predictions should be further confirmed by *in vitro* and *in vivo* experiments, this study highlights meniscus replacement by SFF anatomical scaffolds as a potential alternative to meniscectomy.

## 1 INTRODUCTION

Menisci are wedge-shaped semi-lunar disks present between the weight bearing joint surfaces of the femur and the tibia. Their main functions are to provide stability to the joint, to absorb mechanical shocks, and to assist in the lubrication and in the nutrient supply of the over- and under-lying articular cartilaginous plateaus through the synovial fluid [1, 2]. Only about 20% of the meniscus is supplied with blood in the periphery where it joins to the vascular knee lining [1, 3]. As a result of the avascularity a torn meniscus does not have a satisfying ability to heal itself. In addition, with age, the meniscus starts to deteriorate, often developing degenerative tears. Typically, when the meniscus is damaged, the torn piece begins to move in an abnormal fashion inside the joint causing pain [4].

Even though tears in the meniscus can be treated with different surgical techniques, partial or total meniscectomy are still among the most common solutions to restore the knee functionality [5-7]. However, meniscectomy often leads to articular cartilage breakdown and induces osteoarthritis [5, 8]. This can cause pain and joint impairment [4, 8, 9]. In this respect, partial meniscectomy might be preferred over total meniscectomy to decrease cartilage damage and delay osteoarthritis incidence. Nevertheless, osteoarthritis will still occur [7]. Therefore, alternative methods to restore the joint articulation while keeping menisci functionality are needed. A possible alternative to meniscal removal is the implantation of a tissue engineered meniscus, which might result in the restoration of the original tissue functionalities [2, 10-12]. This tissue engineered construct typically consists of a temporal template – a scaffold – where cells are seeded and can migrate to homogeneously form the desired tissue through the production of extra cellular matrix [13, 14]. Scaffolds are designed to satisfy a number of general requirements, which are critical for the correct tissue regeneration [15-17]. One of the main prerequisites for scaffolds is that they have to withstand applied loads in the implantation site. Therefore, scaffolds are often fabricated to mimic the mechanical behaviour of the tissue to be replaced [15, 18, 19]. Different techniques are nowadays available to manufacture 3D scaffolds with custom-made shapes and tailored mechanical properties [20-23]. Among these, solid free-form fabrication (SFF) technologies offer an undoubted versatility as they can process different biomaterials to generate a porous structure with tailored mechanical properties [24-26]. Furthermore, SFF allows generating anatomical 3D scaffolds from patients’ computer tomography (CT) or magnetic resonance imaging (MRI) datasets [15, 27].

The aim of this study was to fabricate an anatomical 3D meniscal scaffold with porosity and architecture that mimics the mechanical properties of menisci. A porcine meniscal model was considered, since its dimensions and loading are comparable to human menisci [28, 29]. The anatomical 3D scaffolds were experimentally analyzed to tailor their mechanical properties to match those of natural menisci and numerically investigated with finite element analysis (FEA) to determine whether such a construct is – at least from a biomechanical point of view - beneficial for meniscus reconstruction as compared to meniscectomy. Scaffolds were fabricated by 3D fiber deposition (3DF), a versatile rapid prototyping technique that can create structures with interconnected pore networks and allows modelling of the mechanical properties depending on scaffold geometry and architecture [22, 30]. We hypothesized collagen fibers are the extra-cellular matrix (ECM) component mainly contributing with their orientation to meniscus mechanical properties. For this reason, the architecture of the scaffolds was varied by changing the deposition patterns of the fibers comprising the 3DF constructs. Specifically, the deposition pattern was modified from a homogeneously perpendicular architecture to a more radially oriented configuration. This was done in the attempt to mimic the collagen fibers alignment in the meniscus. The alignment of collagen fibers differs throughout the height of the meniscus. In the bottom and top regions the fibers are arbitrarily oriented. In the middle region the fibers are mainly aligned along the curvature of the meniscus. It has been shown that due to this alignment the meniscus is 10 times stiffer in radial compression than in axial compression [4]. Shear stresses are also induced between the collagen fibrils, because the menisci are forced out radially when a compressive axial load is experienced. Due to this orientation, large differences are found in the mechanical properties along the circumferential and the radial direction.

## 2 MATERIALS AND METHODS

### 2.1 Materials

3DF anatomical scaffolds were fabricated with poly(ethylene oxide-terephthalate)-co-poly(butylene terephtalate) (PEOT/PBT), which is normally referred to as aPEOTbPBTc (Isotis S.A., U.S.A.) where a is the molecular weight of the starting PEG blocks used in the copolymerization, while b and c are the weight ratios of the PEOT and PBT blocks respectively. In particular, 300PEOT55PBT45 was used for its better mechanical properties [24, 31]. Scaffolds with solid (SF) and hollow (HF) fibers were considered. HF scaffolds were created by selective leaching as elsewhere described [32]. Blends of PEOT/PBT with Poly(butylmethacrylate-methylmethacrylate) P(BMA/MMA) with a molecular weight of 150 kg/mol, which is selectively soluble in acetone, were used for this purpose (Sigma- Aldrich).

### 2.2 Porcine Knee Meniscus Harvest

Porcine knee menisci were harvested from 6-month old male swines. Once the knee was opened, the menisci and the cartilage plateaus were kept hydrated with phosphate-buffered saline (PBS) (Gibco-BRL). The samples were then stored in PBS and mechanically tested during the same day. Whole lateral and medial menisci and cylindrical samples of 6 mm in diameter by approximately 4 mm in height were tested. Cylindrical meniscal plugs were punched out with a cork drill along the perpendicular axis of the menisci, over their flattest area. Samples were trimmed to ensure parallel surfaces for mechanical testing.

### 2.3 Scaffold fabrication

Anatomical 3D scaffolds were fabricated with a a Bioplotter device (Envisiontec GmbH, Germany), essentially an XYZ robotic arm apparatus, as previously described [24, 30]. Briefly, the polymer was placed in a stainless steel syringe and heated at T = 190 °C through a thermo-set cartridge unit, mounted on the “X”-mobile arm of the plotter. When the polymer reached a molten phase, a nitrogen pressure of 5 Bars was applied to the syringe through a pressurized cap. The anatomical models were obtained by a vivaCT scanner (Scanco, Bassersdorf, Switzerland) with an isotropic voxel size of 20 μm and by MRI datasets of porcine meniscus. The datasets were processed with a CAD/CAM modeling software (Rhinoceros^®^), the final models transferred to the Bioplotter CAD/CAM software (PrimCAM^®^), and plotted layer by layer through the extrusion of the polymer on a stage as a fiber at a deposition speed of 300 mm/min. The produced scaffolds were characterized by the fiber diameter (d1), the fiber spacing within the same layer (d2), the layer thickness (d3) and the configuration of the deposited fibers. By changing these parameters it was possible to change the porosity and the architecture of the scaffold. The nozzle used to extrude polymer fibers were stainless steel Luer Lock hypodermic needles with internal diameter (ID) of 400 μm, shortened to a length of approximately 16.2 mm. The fiber spacing d2 was varied from 1 mm to 1.2 mm, and the layer thickness d3 was set to 200 μm, and 225 μm. This resulted in 3DF scaffolds with a porosity of 70 % and 80 % approximately. The architecture was changed by plotting fibers with 45° and 90° angle steps between successive layers as shown in Table **1**. As the alignment of the collagen fibers differs throughout the height of the meniscus, a “mimicking” scaffold architecture was also considered, where the scaffolds fiber deposition pattern was approximated to the collagenous fibers architecture in the meniscus. In the bottom and top regions, the fibers are arbitrarily oriented. In the middle region, the fibers are mainly aligned along the curvature of the meniscus. This fiber alignment has been proven to result in a 10 -fold stiffness increase in radial compression as compared to axial compression [4]. So for the “mimicking” scaffold, the bottom and top 1 mm of the meniscal scaffold consisted of 0°/45°/90°/135° angle deposition architecture, while the middle part of a 0°/90° angle deposition structure. The 0°/45°/90°/135° architecture was chosen to best approximate the meniscal fiber radial orientation in the top and bottom regions with the angle deposition flexibility offered by the CAM software.

**Fig. (1):**
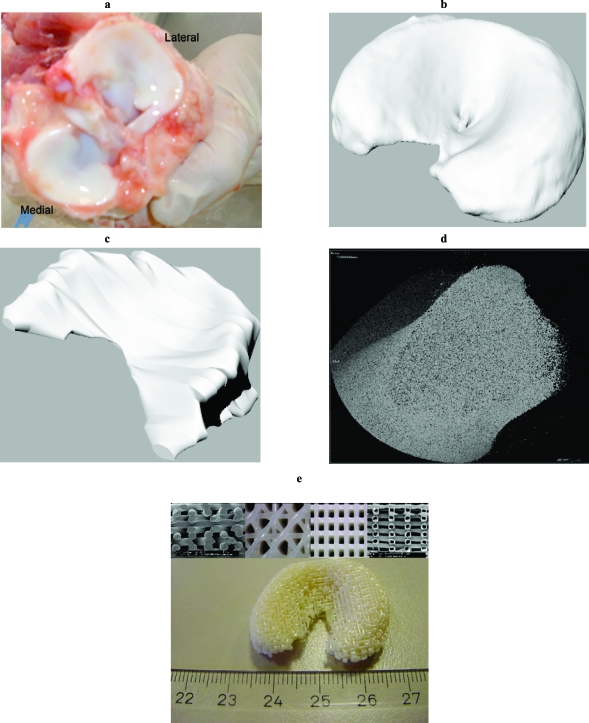
Porcine menisci (**a**) and correspondent models after CT (**b**) and MRI (**c**) scanning. (**d**) 3D reconstruction of a meniscus section. (**e**) Fabricated 3DF anatomical meniscal scaffold with solid and hollow fibers (inserts showing the fiber deposition orientations).

Scaffolds with hollow fibers were also produced by extruding an immiscible 50% w/w blend of 300PEOT55PBT45 and P(BMA/MMA), as previously reported [32]. The blend was deposited at T = 220 °C with a deposition speed of 230 mm/min. The extrusion needle had an ID of 400 μm , and a length of approximately 16.2 mm. The fiber spacing was fixed to 1.2 mm, while the layer thickness to 225 μm, resulting in a structural porosity of 80%. After the meniscal scaffolds were produced, P(BMA/MMA) was selectively leached out in acetone (Sigma-Aldrich) resulting in structures with hollow fibers (see Fig. **1**).

### 2.4 Scaffold Characterization and Mechanical Testing

Anatomical 3DF scaffolds and cylindrical plugs of 6 mm in diameter by 4 mm in height were cored out in the “Z-direction” from rectangular 3D plotted blocks with the same fiber deposition architecture and mechanically tested. The porosity of 3D fiber-deposited scaffolds was calculated as [33]:

(1) $$P=1-\frac{V_{\text{scaffold}}}{V_{\text{cube}}}=1-\frac{\pi }{4}\cdot \frac{1}{\frac{d_{2}}{d_{1}}}\cdot \frac{1}{\frac{d_{3}}{d_{1}}}$$

where P is the scaffold porosity, d1 the fiber diameter, d2 the fiber spacing and d3 the layer thickness, within each different structure.

Since the shape of the anatomical meniscal scaffolds and of the porcine menisci is not uniform, the stress is not distributed evenly. Therefore, the extrinsic compressive stiffness, which is an index of the specimens’ stiffness related to their shape, was calculated [34]. Confined compression tests on whole scaffolds were performed with a testing machine (Zwick, Germany). Confinement was provided by an anatomical set of moulds reproducing the real position of the menisci in the joint. Moulds were custom fabricated by replicating the joint articulation with thermo-setting ceramic plaster. A 5% cyclic sinusoidal strain was applied at a frequency of 1 Hz (n=3). The extrinsic stiffness was calculated as [34]:

(2) $$\text{Extrinsic Stiffness}=E\cdot \frac{A}{h}=\frac{\Delta F}{\Delta L}$$

where E is the intrinsic dynamic stiffness, A is the projected area in compression, and h is the height of the samples, while ΔF and ΔL are the variations of the applied force and of the experienced deformation in the elastic region.

The unconfined equilibrium compressive modulus was also measured on cylindrical plugs of 6 mm in diameter by 4 mm in height by a creep recovery test. A dynamic mechanical analysis (DMA) instrument (Perkin Elmer 7e) was used for this purpose. For each structural and architectural configuration three samples were tested in all the experiments performed. In the case of porcine meniscal cartilage, plugs from both the lateral and the medial menisci were tested. Samples were pre-loaded with a recovery force of 100mN for two minutes. A creep force of 3.5N was applied instantaneously and kept for three minutes, after which the loading condition was returned to the recovery value. The equilibrium modulus was calculated as:

(3) $$E_{0}=\frac{\sigma_{0}}{\varepsilon_{0}}$$

where E_0_ is the equilibrium modulus, while σ_0_ and ɛ_0_ are the stress and strain at equilibrium. The loading conditions were cycled for three times.

### 2.5 Finite Element Analysis

A previously developed finite element analysis (FEA) model was used to determine whether a tissue engineered construct would result in less articular cartilage damage than meniscectomy [35]. The FEA model contains descriptions of fibril reinforcement and osmotic tissue swelling. The strains in the fibril directions and the shear strains along the fibril directions in the knee articular cartilage can be calculated under different loading conditions with this model. Higher fibril and shear strains are believed to indicate cartilage damage, and can induce and enhance cartilage breakdown [36]. Therefore, FEA was used to compare fibril and shear strains in articular cartilage between different loading conditions due to different scaffolds or to meniscectomy.

**Fig. (2):**
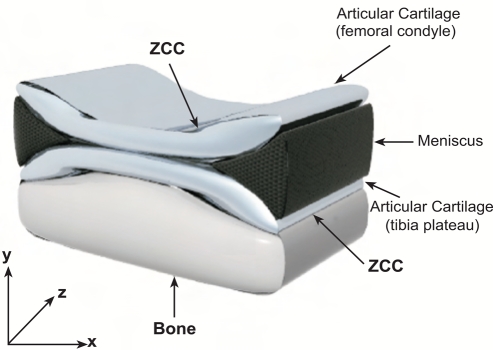
Schematic draw of the axisymmetric finite element model of the knee joint (ZCC=zone of calcified cartilage). Adapted from Wilson *et al.* [30] with permission.

The used numerical model is an axysimmetric representation of a human knee, containing the meniscus and for both femur and tibia x mm of subchondral bone, the zone of calcified cartilage (ZCC), and the deep, middle and superficial zones of the articular cartilage (Fig. **2**). The subchondral bone and ZCC were considered as linear elastic and isotropic materials. The meniscus was assumed transversely isotropic and biphasic, with higher stiffness in the circumferential direction. The isotropic and transversely isotropic models of cartilage originally used by Wilson *et al*. [35] were replaced here with the fibril-reinforced poroviscoelastic swelling (FPVES) model also from Wilson *et al*. [37, 38]. Articular cartilage was assumed biphasic, consisting of (1) a solid phase and (2) a fluid phase. The solid phase consisted of (a) a swelling non-fibrillar part, which contained mainly proteoglycans, and (b) a fibrillar part representing the collagen network. For the non-fibrillar part a compressible neo-Hookean model was used. The swelling behavior was implemented as a combination of osmotic swelling and chemical expansion. The fibrillar part consisted of large primary fibrils and smaller secondary fibrils. Bundles of primary fibrils extended perpendicular from the subchondral bone, splitting up close to the articular surface into fibrils curving to a horizontal course, flush with the articular surface. Each bundle is assumed to split in two directions as in the Benninghoff model [39]. A network of secondary fibrils was represented as a random, homogeneous 3D network. Collagen fibrils were assumed to be viscoelastic [37].

Each simulation consisted of three phases. The first phase was free swelling to gain equilibrium. In the second phase a force of 588N was applied for one second to a bottom node, which was tied to the other bottom nodes. The third step consisted in a consolidation phase of 20 seconds. In Table **2** the material parameters that were used for the meniscus in healthy conditions are given. The model was implemented in Abaqus v6.3 finite element software (Hibbitt, Karlson, Sorensen, Inc. Pawtucket, RI, USA).

The parameters as in Table **2** were changed to determine the fibril and shear strains when a scaffold is placed in absence of the meniscus in the model. The material properties of the meniscus were adapted to a linear isotropic behaviour, since the polymer used to print the scaffolds (300PEOT55PBT45) shows linear isotropy [40]. The equilibrium young’s modulus was considered for all different scaffolds as the equilibrium modulus of 300PEOT55PBT45 (34 MPa), while the Poissons’ ratio differed for the different architectures considered and accounted for the different anisotropic behaviour resulting from the scaffold’s architectures. The Poissons’ ratio were calculated by a micro-optical method, as previously described (Table **3**) [22]. No swelling was accounted for the polymer, as this normally falls within 6% for 300PEOT55PBT45 [24]. For these scaffolds the exact permeability *in vivo* has not been calculated. Different simulations were done in which the permeability was 10 and 100 times increased and 10 and 1000 times decreased. It was also calculated in which range the permeability lies. For the higher possible range of permeability, the highest porosity with the lowest and highest volume of entrapped wetting liquid in the medium was considered in the calculation. Similarly, for the lowest possible range of permeability, the lowest porosity with the lowest and highest volume of entrapped wetting liquid in the medium was considered. The mean of the calculated values was taken, resulting in a hydraulic permeability k of 1.26*e-2*10^-15^ m^4^/Ns. So the scaffold is assumed to be100 times more permeable than the meniscus.

## 3 RESULTS

### 3.1 Scaffolds Characterization and Mechanical Testing

**Fig. (3):**
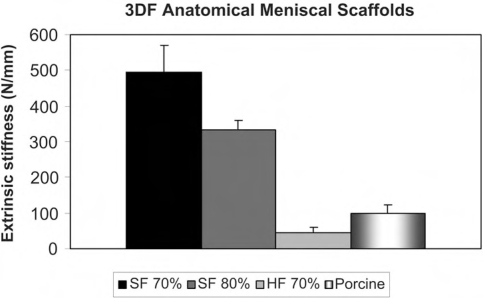
Extrinsic stiffness of 3DF anatomical mensical scaffolds.

Anatomical 3DF scaffolds were successfully fabricated with solid and hollow fibers from both CT and MRI datasets (Fig. **1**). A better resolution in the meniscal model was found for CT datasets. Therefore, 3DF scaffolds for mechanical testing were produced only from the CT derived model. The extrinsic stiffness of 3DF scaffolds with solid (SF) and hollow (HF) fibers is shown in (Fig. **3**) and compared to the extrinsic stiffness of porcine menisci. With increasing the porosity of SF 3DF scaffolds, the extrinsic stiffness decreased. In particular, the extrinsic stiffness of SF scaffolds varied from 495.07 ± 76.26 N/mm to 333.22 ± 26.16 N/mm when scaffold porosity increased from 70% to 80%. When HF scaffolds were tested, the extrinsic stiffness was measured as 43.94 ± 14.71 N/mm for scaffolds with a structural porosity of 70% (structural porosity doesn’t take into account the added porosity of the hollow cavities). The porcine meniscal extrinsic stiffness was 98.52 ± 25.89 N/mm.

**Fig. (4):**
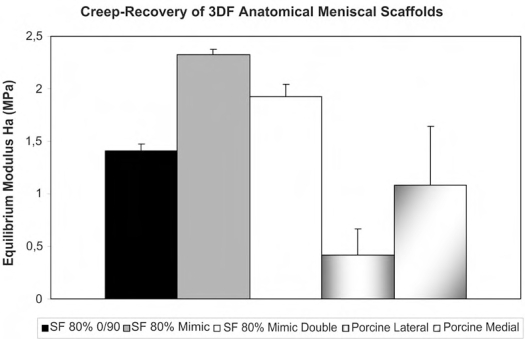
Equlibrium modulus of 3DF anatomical meniscal scaffolds.

The equilibrium modulus E_0_ of SF 3DF scaffolds varied with different fiber architectures, while the porosity was maintained constant (Fig. **4**). Specifically, E_0_ was 1.41 ± 0.07 MPa for a 0/90 architecture, 2.33 ± 0.05 MPa for a mimicking architecture, and 1.92 ± 0.12 MPa for a mimicking architecture were the angle deposition between subsequent fibers was changed every two layers. The porcine menisci had an equilibrium modulus of 0.42 ± 0.25 MPa or of 1.08 ± 0.56 MPa when lateral or medial menisci were considered respectively.

### 3.2 Numerical Simulation

The influence of changes in scaffold permeability on articular cartilage maximal fibril and shear strain is shown in Fig. (**5**). It can be seen that the effect on cartilage is almost negligible except when the permeability is increased 100 times with respect to the value considered in the normal (healthy) situation; in this case the strains are lower. As the Poisson’s ratio of the scaffolds varied with varying their architecture (Table **3**), the model was adapted accordingly. Fig. (**6**) depicts the results from the numerical simulations when 3DF scaffolds with different architecture are considered to replace the meniscus as compared to meniscectomy. The strains in the articular cartilage are comparable for the different scaffolds analyzed. All of the scaffolds result in less strain than meniscectomy.

**Fig. (7):**
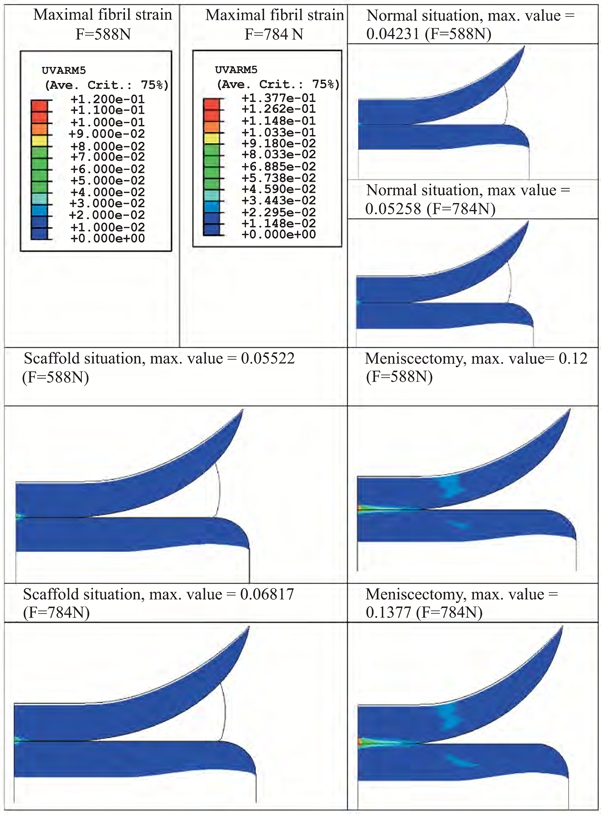
Influence of applied load amplitude on articular cartilage maximal fibril strain.

**Fig. (8):**
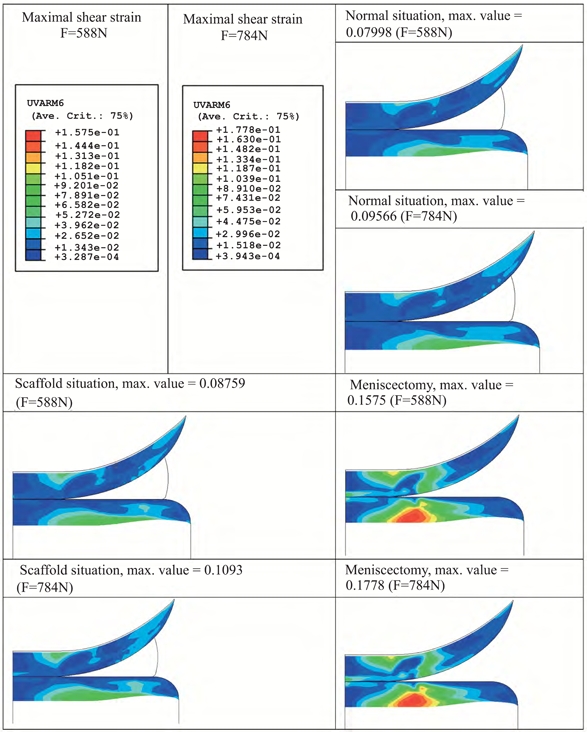
Effect of applied load amplitude on articular cartilage maximal shear strain.

The effect of a 30% load increase, from 588 N to 784 N, was also assessed in physiological conditions, or by replacing the meniscus with a 3DF scaffolds (0°0°/90°90° architecture), or after meniscectomy. The maximal fibril strain value increased by 30% when a meniscus was replaced by a scaffold compared to the healthy situation (Fig. **7**). However, a 183% to 162% increase for an applied load of 588 N and 784 N was respectively found when meniscectomy was chosen as a treatment. The maximal shear strain value increased 9% for 588 N and 14% for 784 N when a minisucus was replaced by a scaffold with respect to the maximal value in healthy situation (Fig. **8**). The increase was 97% and 86% after meniscectomy.

The influence of the loading time was also analyzed. An increase in loading time from 1 second up to 10 seconds resulted in an increase of 2% for the maximal fibril and shear strain for both the healthy situation and the scaffold, as expected. In all cases the maximal fibril strain was approximately 30% higher while the maximal shear strain was about 10% higher than in healthy conditions.

## 4 DISCUSSION

Rapid prototyping techniques allow the fabrication of scaffolds with a controlled pore network and with a custom made shape. In this study, anatomical 3D scaffolds for meniscal replacement have been fabricated with matching natural menisci biomechanical properties and their beneficial use numerically evaluated in terms of articular cartilage damage. Scaffolds were modelled from CT or MRI datasets of a porcine meniscus, which was chosen as a testing model because of its similarity with humans [28, 29, 41]. From a processing point of view, CT datasets were found to be more accurate and precise than MRI. The difference in the final structure can be associated to the different resolution of CT and MRI. The opacity of the porcine meniscal tissue due to the high water content may be responsible for the lower resolution of the final MRI datasets. This was in part improved by using a contrast fluid. The accuracy losses may also have been caused by the transfer of the MRI image datasets to the processing software to create the CAM file for the final scaffold fabrication. Therefore, CT seems to be a more efficient technique when highly detailed anatomical scaffolds are desired for the reconstruction of tissues with an intrinsic degree of opacity.

The mechanical properties of the meniscal 3DF scaffolds were examined in terms of their extrinsic stiffness and equilibrium modulus. Such stiffness was chosen to consider the overall mechanical response of the analyzed shaped structures as also reported by Turner *et al.* [34]. The extrinsic stiffness and equilibrium modulus of porcine menisci could be mimicked by modulating the pore network size and architecture of 3DF scaffolds. The modulation of mechanical properties to tailor scaffold structures for custom made applications is important to ultimately apply tissue engineered constructs in the clinics for specific patients [15, 42, 43]. In fact, it has been recently shown that cells are capable of sensing the mechanical rigidity in their surrounding at different scales [44-47]. When the stiffness of scaffolds or matrices where cells were seeded matches the stiffness of the natural tissue to regenerate, the final constructs were characterized by enhanced cell differentiation into the proper lineage and by a higher extracellular matrix production [47-49]. Different scaffold fabrication techniques can generate structures with a certain control over the mechanical properties of the resulting cellular solids [23, 50-52]. However, rapid prototyping technologies offer undoubtedly the possibility to more precisely control not only their mechanical properties, but also their pore network architecture and their custom shape [15, 53-55]. Scaffolds with simplified shapes were also fabricated and mechanically tested. The extrinsic stiffness of scaffolds with different shapes but same porosity and pore architecture resulted in different mechanical response (data not shown). From these findings it appears that the shape of the scaffolds to be implanted also has an important influence on their mechanical behaviour.

The numerical analyses showed that the strains experienced by the knee-joint articular cartilage are higher after meniscectomy than when the meniscus is replaced by one of the examined scaffolds. As expected, the strains in a physiological situation are the lowest. Since it has been proposed that higher strains induce more damage in articular cartilage, we may conclude from this simulation that from a mechanical point of view replacing the meniscus with an anatomical scaffold would better resemble physiological conditions than meniscectomy. Highly permeable scaffolds (permeability 1000 times higher than meniscus) seemed to have a slightly more beneficial effect on the over- and under-lying cartilagineous plateaus. However, such a high permeability implies high porosity and large pore size, which might have a counter effect on cell entrapment efficiency if these scaffolds are used for tissue engineering applications. Furthermore, an increase in porosity would also determine a correspondent decrease in the mechanical properties, thus affecting anyhow cell differentiation [47] and resulting in more strains applied to the articular cartilage. To some extent, this counteracts on the beneficial effect of increasing the scaffold permeability. Thus, the permeability was maintained constant to the value estimated in Table **2** for the other simulations. Furthermore, it has been shown that the more porous is a scaffold structure, the rougher is the surface, which ultimately results in poor frictional properties [2]. Although this might be a critical issue for the proper biomechanical function of the anatomical scaffolds, the surface of the fibers comprising the specific scaffolds treated in this study are rather smooth despite any variation in porosity. Still, further analysis on the friction change with varying the scaffolds’ architecture and structural porosity should be considered to better address the effective use of such scaffolds as meniscal replacements.

Scaffolds with matching mechanical properties but different pore architecture did not considerably influence the strains applied on the articular cartilage surfaces. Therefore, the pore network architecture analyzed here seems not to be a determinant factor in terms of the mechanical functional restore of the articulation. An increase in the amplitude (Figs. **7** and **8**) or in the duration of the applied load corresponded to an increase in the strains experienced by the femoral and tibial cartilage. Yet, this increase sheds further light on the beneficial effect of replacing the meniscus with a scaffold than simply removing it. Specifically, the presence of a 3DF anatomical scaffold corresponded to a 5-fold and 9-fold decrease in the strains as compared to meniscectomy, and therefore would lead to less damage in the articular cartilage plateaus. This reduction indicates that the beneficial effect of replacing the meniscus with a scaffold, rather than removing it, has additional pronounced effects in preventing damage due to high-impact overloading.

The present results are also intriguing in the context of recent experimental [47, 56] and numerical [57-59] reports, where it was shown that matching the mechanical environment of a specific tissue would result in proper cell differentiation and tissue formation. However, it has still to be analyzed whether the pore network architecture plays a major role on *in vitro* tissue formation. *In vivo* experimental data to verify the prediction by the numerical simulations that scaffold porosity is a key parameter in the design of meniscus biomaterial implants is currently lacking and needs to be the focus of future studies. In this respect, a major challenge is represented by not only regenerating the cartilaginous tissue, but also the vasculature connections of menisci to the knee lining. The numerical analysis here considered to evaluate the biomechanical effect of anatomical 3D scaffolds as menisci replacements may also be further optimized. The model used is axysimmetrical and simplifies the real 3D geometry of articular and meniscal cartilage. Moreover, the damping effect of synovium fluid is not considered in its implementation. Yet, FEA is a useful tool to identify in a preliminary evaluation the importance of particular characteristics for the design of a scaffold for tissue replacement or as templates for tissue engineering applications.

## 5 CONCLUSIONS

Anatomical 3DF scaffolds with matching mechanical stiffness have been fabricated and characterized for the replacement of damaged menisci. Numerical simulations showed that the replacement of natural menisci with these constructs would result in a significant improvement of the strain and stress distribution on the over- and under-lying articular cartilage as compared to meniscectomy, the current most applied clinical procedure. The beneficial effect of these scaffolds might be further considered as a possible alternative to treat meniscal damages and replacements.

## Figures and Tables

**Fig. (5):**
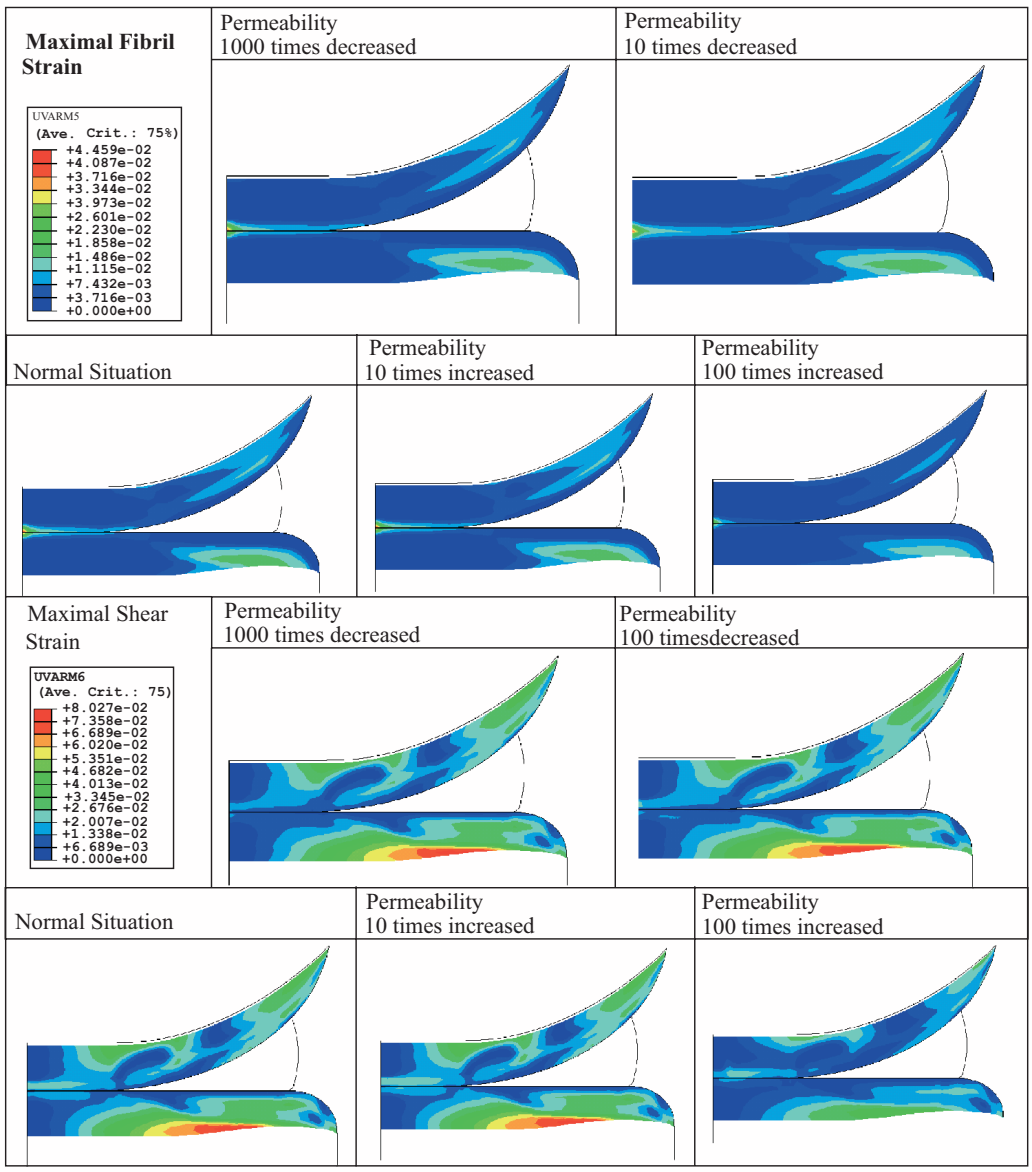
Effect of permeability on articular cartilage maximal fibril strain.

**Fig. (6):**
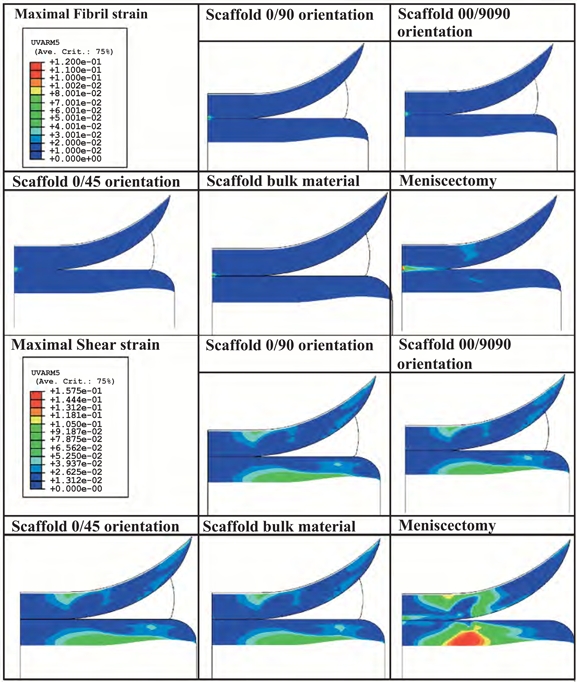
Influence of scaffold architecture on articular cartilage maximal fibril strain.

**Table 1:** Scaffold and Pore Network Architecture

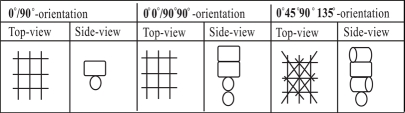

**Table 2:** Mechanical Parameters Considered During the Numerical Simulations

	Bone	ZCC	Meniscus
Young’s Modulus *E_1_, E_2_* [MPa]	400	8	0.075
Young’s Modulus *E_3_* [MPa]	-	-	100
Poisson’s ration *v*_12_ [-]	0.3	0.49	0.5
Poisson’s ration *v*_13_ [-]	-	-	0.0015
Shear Modulus *G* [MPa]	-	-	0.025
Permeability coefficient *k_0_* [10^-15^ m^4^/Ns]	-	-	1.26
Solid volume fraction *n^s^* [-]	-	-	0.75

**Table 3:** Poisson’s Ratios of the 3DF Scaffolds with Varying their Architecture

Architecture	Paission Ratio
0°/90°	0.321
0°0°/90°90°	0.415
0°/45°	0.442
Bulk material	0.48
